# Unexploited opportunities for phage therapy

**DOI:** 10.3389/fphar.2015.00180

**Published:** 2015-09-07

**Authors:** Hugo Oliveira, Sanna Sillankorva, Maia Merabishvili, Leon D. Kluskens, Joana Azeredo

**Affiliations:** ^1^Laboratório de Investigação em Biofilmes Rosário Oliveira, Centre of Biological Engineering, University of MinhoBraga, Portugal; ^2^Laboratory for Molecular and Cellular Technology, Queen Astrid Military HospitalBrussels, Belgium

**Keywords:** bacteriophage therapy, bacterial pathogens, infectious diseases, novel lytic bacteriophages, antibiotic resistance

## A surprising twist on bacteriophage therapy

Bacterial infections have always been a threat to human health. The first available systemic antibiotics, in early twentieth century, were initially seen as truly miraculous drugs, providing a powerful tool to combat bacterial infections and increasing human life span. Deluded by their exceptional antimicrobial properties, they quickly fell in trivial use to fight even the most common types of infections, many of non-bacterial nature. This indiscriminate use of antibiotics allowed bacteria to develop defense systems to become less prone to antibiotics - multiresistant-superbugs, a development that Sir Alexander Fleming already anticipated when he gave his Nobel lecture in 1945.

Bacteriophages (phages) are natural predators of bacteria that specifically parasite bacteria to replicate, and were discovered in the early part of the twentieth century, independently by Twort (1915) and d'Herelle (1917) (Summers, 2005). The ability to kill bacteria was soon therapeutically explored by d'Hérelle and his followers while fighting various bacterial infections around the world, such as the bubonic plague in Southeast Asia, dysentery in France, and cholera in India. Phage therapy was extensively used to treat infectious diseases during World War II too (Summers, 2001). However, insufficient knowledge of phage biology, low quality control of phage preparations and lack of solid scientific evidence of therapeutic successes led to phage failure over the newly discovered antibiotics till the second half of the twentieth century.

The alarming emergence of antibiotic resistant bacteria is considered the greatest threat to human health of the new millennium by both the Infectious Diseases Society of America (IDSA) and the European Society of Clinical Microbiology and Infectious Diseases (ESCMID) in addition to many other health-caring organizations worldwide (El-Tahawy, 2004; Rice, 2009). With this new reality, scientific community and business players have revived their interest in phages as an alternative therapy. The ability of phages to kill antibiotic-resistant bacteria allied with their ubiquitous nature, high specificity (minimal disruption of normal flora), prevalence in human gastrointestinal tract, self-replication ability at the infection site, and low inherent toxicity qualifies them probably as the most “safe” and “green” technology available for clinical application. Several studies are currently providing convincing evidence regarding the safety and efficiency of phage therapy in animals and humans (Sulakvelidze et al., 2001; Sillankorva et al., 2012), with recent appearances of new phage companies and commercial phage-based products (Thiel, 2004; Housby and Mann, 2009).

This opinion article reports on a number of bacterial pathogens for which virulent phages can still be isolated but till now have been neglected due to various reasons listed below.

## Finding the next phage—the road ahead for phage therapy

With the renewed excitement and the successive regulatory affairs opening for phage application, the possibility to use phage products designed “a la carte” to treat therapeutically every bacterial infections seems endless (Brüssow and Hendrix, 2002; Rohwer, 2003; Pirnay et al., 2010). In recent years, FDA has approved phage preparations to be used as food additives (e.g., Listex, EcoShield) (FDA, 2006). Also, the World Medical Association in its Declaration of Helsinki provides a way for phage therapy, as a representative of non-conventional type of medicine, to be used complying the ethical principles for medical research involving human subjects (World Medical and Association, 1964/2013). European regulatory authorities nowadays consider phages as biological agents which accordingly require European trials to follow the current biological medicinal product guidelines, while trials in the USA need to be complied with the guidelines of the division of vaccines and related product applications. However to date, no phage therapy-specific guidelines have been published by any of these authorized agencies.

From a therapeutic perspective, strictly lytic phages are required to lyse pathogenic bacteria and persist by releasing virion progeny that can migrate to other sites in the body. The currently revived attention toward phage therapy is mostly due to the worldwide emergence of antibiotic-resistant bacterial species, known before as either opportunistic or rare pathogens. A good example for this phenomenon is *Acinetobacter baumannii*: known as a low-grade pathogen in the 70 s (Joly-Guillou, 2005) and currently listed as one of the six top-priority microorganisms by IDSA (Boucher et al., 2009). However, despite the growing therapeutic appreciation of phages, there are still many human bacterial pathogens, according to the approved list of biological agents (Health Safety and Executive, 2013), for which lytic phages have not yet been found, including representatives of important biosafety level 3 bacteria, such as *Rickettsia, Ehrlichia*, and *Coxiella*. Although these bacteria are not an eminent public health hazard, low infectious doses of them can cause severe to fatal diseases (ehrlichiosis, epidemic typhus, and Rocky Mountain spotted fever and Q fever). Within the same safety category, several *Mycobacteria* species, like *M. africanum*, and *M. leprae*, the latter etiological agent of Hansen's disease (leprosy), have not yet been targeted by phage therapy either. Depending on the type, nature and source of infection, we envisage several novel phage applications.

### Wound and skin infections

Wounds and burns cause breaches in the natural protective skin barrier, making it susceptible to infection. Several skin conditions are caused by *M. marinum, M. szulgai*, or *Treponema pertenue*. In case of chronic wounds, *Actinomycetes* and *Actinomadura* are responsible for a subcutaneous subacute-to-chronic actinomycosis and mycetoma, respectively. Topical solutions, such as ointments, creams or lotions containing lytic phages could be incorporated in cosmetic and pharmaceutical industry products to treat these conditions. For purulent chronic wounds, phages can be applied in the course of wound irrigation, either by injection into/around the wound, by soaked bandages, or by impregnation into biodegradable polymer wound dressings with a time-release manner. The major challenge in treating skin infections caused by intracellular bacteria including *Mycobacterium* spp. is to develop appropriate strategies for delivery of bacteriophages into mammalian cells (Chacko et al., 2012). Nonetheless several intracellular pathogens have a temporary extracellular living form where they can be more easily tackled by phages; this is the case of *M. ulcerans*, against which phage therapy has been proven efficient (Trigo et al., 2013).

### Respiratory, gastrointestinal, and urogenital tract infections

The bacterial colonization of mucous membranes provides a potential starting point for more severe and sometimes life-threatening infections. Phages specific for *Klebsiella pneumonia, A. baumannii, Pseudomonas aeruginosa*, and *Staphylococcus aureus* have been extensively studied. However, there are other pathogens involved in severe respiratory infections still uncovered by phage therapy: *Haemophilus influenzae* (bronchitis), *Corynebacterium diphtheria* (diphteria), and *Porphyromonas* spp. are responsible for upper respiratory tract infections; *Helicobacter pylori, M. simiae*, or *Nocardia* spp. infect the lower respiratory tract as well as *Fluoribacter bozemanae, Mycoplasma pneumoniae*, or *Ureaplasma urealyticum* presenting etiological agents of pneumonia. In this type of infections, effective phage administration can be done via the respiratory route by aerosol inhalation. No lytic phages are available to fight enteric infections caused by *Salmonella arizonae, Porphyromonas* spp., *Providencia alcalifaciens, Hafnia alvei*, or *Serpulina* spp. Gastrointestinal phage treatment offers advantages over antibiotics by the reduced disruption of the gut flora. However, phages are sensitive to the low pH of the gastrointestinal tract, therefore should be encapsulated when delivered orally, as already tested *in vivo* studies in feedlot cattle *with Escherichia coli* O157:H7 phages (Callaway et al., 2008). Concerning urogenital tract infections, there are no known lytic phages for *Legionella pneumophila* and *Proteus penneri* or *P. rettgeri* and *Citrobacter koseri* species responsible for nosocomial urinary tract infections. Treating urogenital infections with phages can be similar to conventional methods involving antibiotic applications (e.g., intramuscular/subcutaneous injections or irrigation of bladder). In this context, the use of indwelling hydrogel-coated catheters combined with phages may be a very attractive approach for managing these particular bacterial infections in hospitals. As far as bacterial vaginosis is concerned, *Gardnerella vaginalis* lytic phages could be an interesting therapeutic option when impregnated into tampons or personal hygiene products. Additionally, phage therapy can potentially target bacteria causing sexually transmitted infections, such as chancroid (*Haemophilus ducreyi*), chlamydia (*Chlamydia trachomatis*), gonorrhea (*Neisseria gonorrhoeae*), syphilis (*Treponema pallidum*), and granuloma inguinale (*Klebsiella granulomatis*).

### Bacteremia and septicemia

The presence of viable bacteria in the human circulating blood, known as bacteremia, can result in systemic infections. Common localized infections, like pneumonia, urinary tract or bladder and skin infections may become systemic too. Relevant pathogens causing systemic infections are for example: *Francisella* spp. (tularemia), *Leptospira interrogans* (leptospirosis), *Brucella canis* (brucellosis), *Ehrlichia sennetsu* (monocytic ehrlichiosis), *Rickettsia* spp. (e.g., typhus, rickettsialpox, Rocky Mountain spotted fever), *Treponema* spp. (syphilis and yaws), *Peptostreptococcus anaerobius* (endocarditis) or *Clostridium tetani* and *botulinum* (tetanus and botulism). Intravenous and parenteral delivery of phages are considered the most routinely-employed methods of administration, supplying sufficient numbers of phages to eliminate bacteria from the bloodstream. Transdermal delivery also offers a potential means of overcoming many problems associated with systemic phage administration.

## (UN)expected challenges

**Figure 1:**
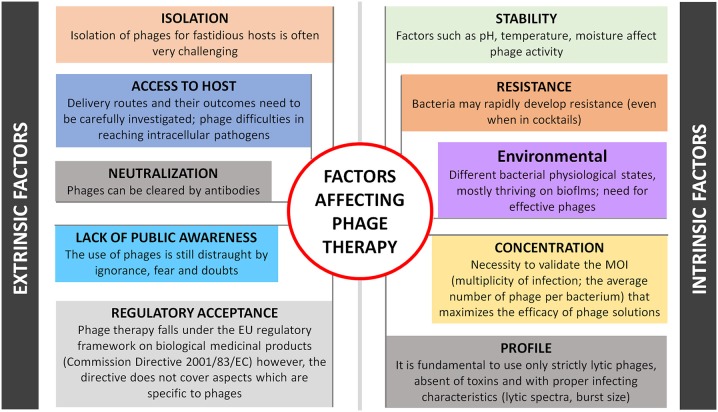
**Schematic representation of factors that limit phage therapy**.

One can anticipate several challenging factors of therapeutic and biotechnology development of phages, as depicted in Figure 1. Intrinsic-driven factors are not discussed here but refer to phage profile (e.g., virulent or temperate nature, specificity, burst size), stability (e.g., pH, temperature), concentration (ideal multiplicity of infection is phage dependent), environmental factors and its inherent ability to induce bacterial resistance. These limitations have been extensively discussed elsewhere (Loc-Carrillo and Abedon, 2011). External factors can be considered more problematic as they are difficult to anticipate and resolve, such as: (i) Phage isolation, difficulties can be expected in the isolation of some of the phages, already a demanding task that can become even more challenging for phages targeting fastidious hosts as their availability and likelihood of being found in the environment are relatively low; (ii) Phage toxicity, although considered inherently non-toxic, due to its nucleic acid and protein nature, phages are produced in the presence of bacterial hosts, therefore a careful design of the downstream processes is required to avoid the presence of any bacterial toxins in the phage product (Merabishvili et al., 2009). The factor of releasing endotoxins after bursting bacterial cells inside the human body may limit phage treatment of Gram-negative systemic infections too, however the same challenges exist for some antibiotics and more detailed studies will be needed for each specific phage and bacterial host; (iii) Phage neutralization, some studies have shown that phages are removed by the reticuloendothelial system and inactivated by the development of neutralizing antibodies like most pharmaceuticals that interact with the body's immune system (Westwater et al., 2003; Lusiak-Szelachowska et al., 2014). Although the risks are minimal because of low speed of these processes and non-dependence of successful outcome on anti-phage activity of human immune system (Lusiak-Szelachowska et al., 2014), the delivery of less-immunogenic phages either with proper nanocarriers (e.g., liposomes) or by engineering them to have non-immunogenic and biocompatible peptides on their surface (e.g., polyethylene glycol molecules) are considered; (iv) Phage access to host; the choice of the delivery system plays a key role in the success of phage therapy. Recent advances in phage therapy show that targeted delivery has been more successful for localized infection treatment, while for systemic infections the parenteral route is recommended (Ryan et al., 2011). Treatment of intracellular bacterial infections remains the main challenge for medical care. As mentioned above, obligatory or facultative intracellular bacterial pathogens either reproducing themselves and thriving in cells, such as *M. leprae, Chlamydia, Ehrlichia*, and *Rickettsia* or taking transient refuge therein are shielded from many antimicrobials, hence phage therapy may fail too. Finally, and probably the most important barriers in phage therapy are still (iv) Regulatory acceptance and (v) Lack of public awareness. It is not clear yet, which is the best regulatory framework for phase therapy: can phages be considered medicinal products, biological medicinal products or advanced medicinal products, according to Directive 2001/83/EC. Several arguments recently debated by experts representing different stakeholder groups, fit phage therapy partially (but not totally) in every possible definition. This turns regulation of phage products currently difficult to achieve, unless a dedicated European legal framework is created (Verbeken et al., 2014). This “marketing” authorization should also contemplate legislation either for a standard phage-based product or for more specific, tailor-made phage preparations. Overall, the uncertainty of phage-specific regulatory guidelines along with the patentability difficulties, hurdles the potential of pharmaceutical investments. This is practically critical in the Western world, where there is also a low awareness of the potential of phage therapy by large part of medical society. Besides exploiting various therapeutic applications, a tremendous effort is still needed in phage therapy research and on the regulatory side, to bring phages from the bench to the patient's bedside.

### Conflict of interest statement

The authors declare that the research was conducted in the absence of any commercial or financial relationships that could be construed as a potential conflict of interest.
